# Allergic diseases attributable to atopy in a population sample of Asian children

**DOI:** 10.1038/s41598-021-95579-2

**Published:** 2021-08-06

**Authors:** Chao-Yi Wu, Hsin-Yi Huang, Wen-Chi Pan, Sui-Ling Liao, Man-Chin Hua, Ming-Han Tsai, Shen-Hao Lai, Kuo-Wei Yeh, Li-Chen Chen, Jing-Long Huang, Tsung-Chieh Yao

**Affiliations:** 1 Division of Allergy, Asthma, and Rheumatology, Department of Pediatrics, Chang Gung Memorial Hospital, 5 Fu-Hsin Street, Kweishan, Taoyuan Taiwan; 2 Institute of Environmental and Occupational Health Sciences, National Yang-Ming University, Taipei, Taiwan; 3 School of Medicine, Chang Gung University College of Medicine, Taoyuan, Taiwan; 4 Community Medicine Research Center, Chang Gung Memorial Hospital At Keelung, Keelung, Taiwan; 5 Department of Pediatrics, Chang Gung Memorial Hospital At Keelung, Keelung, Taiwan; 6 Division of Pediatric Pulmonology, Department of Pediatrics, Chang Gung Memorial Hospital, Taoyuan, Taiwan; 7 Department of Pediatrics, New Taipei Municipal TuCheng Hospital, 6 Sec. 2 Jinchen Road, Tucheng District, New Taipei, Taiwan

**Keywords:** Immunology, Biomarkers, Diseases

## Abstract

The proportion of allergic diseases attributable to atopy remains a subject of controversy. This study aimed to estimate the population risk of physician-diagnosed asthma, rhinitis and eczema attributed to atopy among a population sample of Asian school-age children. Asian children aged 5–18 years (n = 1321) in the Prediction of Allergies in Taiwanese CHildren (PATCH) study were tested for serum allergen-specific immunoglobulin E. Physician-diagnosed asthma, rhinitis and eczema were assessed by a modified International Study of Asthma and Allergies in Childhood questionnaire. Atopy was defined as the presence of serum allergen-specific immunoglobulin E. In this population-based study, 50.4% of the subjects with asthma, 46.3% with rhinitis, and 46.7% with eczema were attributable to atopy. The population attributable risk (PAR) of atopy for three allergic diseases was higher in adolescents (asthma, 54.4%; rhinitis, 59.6%; eczema, 49.5%) than younger children aged less than 10 years (asthma, 46.9%; rhinitis, 39.5%; eczema, 41.9%). Among the seven allergen categories, sensitization to mites had the highest PARs for all three allergic diseases (51.3 to 64.1%), followed by sensitization to foods (asthma, 7.1%; rhinitis, 10.4%; eczema 27.7%). In conclusion, approximately half (46.3 to 50.4%) of Asian children in Taiwan with allergic diseases are attributable to atopy.

## Introduction

The prevalence of allergic diseases, including asthma, rhinitis and eczema, is increasing worldwide^1–3^. Children with allergic diseases may have significantly impaired quality of life and higher healthcare utilization, representing a substantial health burden^4–8^. A clear understanding of the spectrum of etiologies contributing to allergic diseases may lead to improved strategies for prevention and management of these conditions.

Atopy, defined as the genetic propensity to develop immunoglobulin E (IgE) antibodies in response to exposure to allergen, is a well-known risk factor for allergic diseases^9,10^. Most treatment and prevention strategies nowadays thus focused on mechanisms of allergic sensitization and emphasized on the importance of allergen avoidance. However, some physicians have argued that the proportion of allergic diseases attributable to atopy may have been overestimated, challenging the concept that there are allergic and non-allergic forms of asthma, rhinitis and eczema^10–13^. However, the exact proportion of allergic diseases truly attributable to atopy remains a subject of debate.

Previous studies have demonstrated ethnic differences in the prevalence of atopy as well as allergic diseases^14,15^. Although previous studies in Western countries suggested that 4 to 63% of asthma cases^10,11,16–18^, 37.3 to 80.7% of rhinitis cases^10,12^ and 32% of eczema cases were attributable to atopy^10^, there is limited data available on the proportion of allergic diseases attributable to atopy among Asian children. The objective of this study was to estimate the population risk of physician-diagnosed asthma, rhinitis and eczema attributed to atopy among a population sample of Asian school-age children in Taiwan.

## Methods

### Study subjects

**Figure 1:**
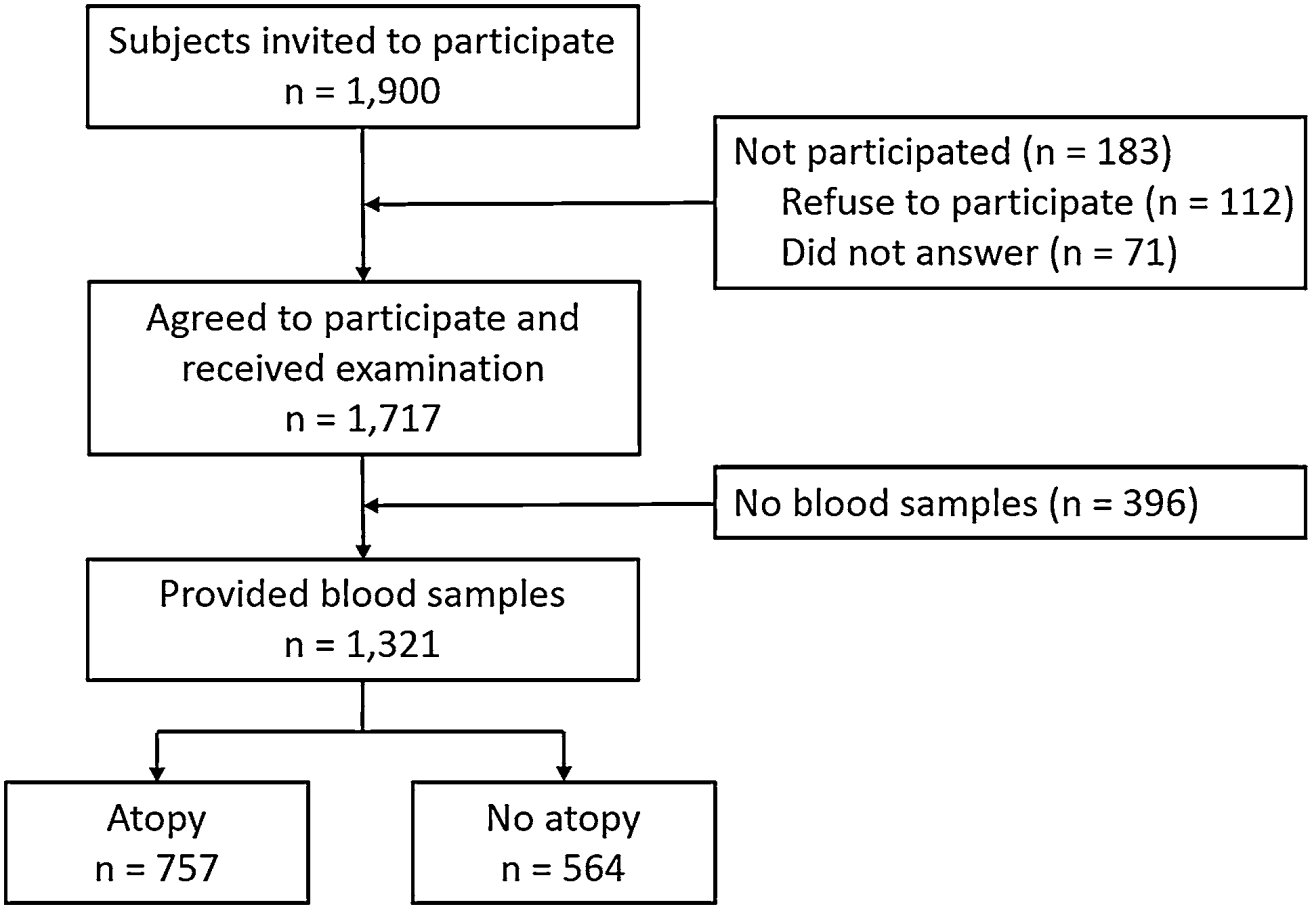
Schematic presentation of the recruitment process of the study subjects.

The schematic presentation of the recruitment process of study subjects is presented in Fig. 1. Study participants were recruited from a population-based cohort of 5351 children (2616 boys, 48.9%; age, 10.4 ± 2.9 years), participating in the Prediction of Allergies in Taiwanese CHildren (PATCH) study^3,19–24^. Among the subjects, 1900 children were randomly invited and 1717 of them agreed to participate the study. Blood samples were successfully obtained from 1321 children to determine allergen-specific IgE. Parents of the recruited subjects answered pre-designed questionnaires modified from the International Study of Asthma and Allergies in Childhood (ISAAC) questionnaire^25^, regarding subject’s general health information, demographic data, and questions on clinical symptoms and diagnosis of allergic diseases. There were no significant differences between the 1321 study subjects and the original 5351 cohort members in terms of gender, age, and prevalence of allergic diseases. The study was approved by the Institutional Review Board of Chang Gung Medical Foundation (96-0370B) and written inform consents were provided by the parents of each subject. All experiments in this study were performed in accordance with the relevant guidelines and regulations.

### Allergen-specific serum immunoglobulin E

Atopy was defined as positive Phadiatop Infant test result (≧ 0.35 PAU/L) (Phadia, Uppsala, Sweden), a commercial assay to detect allergen-specific IgE against a mix of common inhalant and food allergens^26^. Allergen-specific IgEs against 40 common allergens divided in 7 categories were measured by an automated microfluidic-based multiplexed immunoassay system (BioIC^TM^ Allergen-specific IgE Detection Kit- AD40 Panel; Agnitio Science and Technology, Hsinchu, Taiwan). As previously reported^27^, the allergen-specific IgEs includes: (1) mites: Dermatophagoides pteronyssinus, Dermatophagoides farinae, and Blomia tropicalis; (2) animals: dog dander, cat dander, chicken feather and skin, and duck feather and skin; (3) cockroaches: German cockroach and Oriental cockroach; (4) mold: Candida albicans, Cladosporium herbarum, Aspergillus fumigatus, Penicillium notatum, and Alternaria alternata; (5) pollen: timothy grass, bermuda grass, ragweed, mugwort, and goldenrod; (6) foods: cow’s milk, goat’s milk, egg white, egg yolk, crab, shrimp, codfish, salmon, blue mussel, soybean, wheat, potato, peanut, almond, garlic, cheese, baker’s yeast, kiwi, tomato, and carrot; and (7) latex. However, latex was excluded from the analysis of the estimate for population attributable risk (PAR) because its low prevalence resulted in non-convergence of the model^28^.

### Definitions of allergic diseases

This study evaluated 3 allergic diseases, including asthma, rhinitis and eczema. The diagnosis of allergic diseases were assessed using the modified ISAAC questionnaire^25^. Cases were subjects whose parents provided affirmative answers to the questions, ‘‘Has a doctor ever told you that you had asthma?’’, ‘‘Has a doctor ever told you that you had allergic rhinitis?’’ or ‘‘Has a doctor ever told you that you had eczema?’’.

### Statistical analysis

Differences in the prevalence of subject characteristics were analyzed using Chi-squared test or Student’s t-test. Logistic regression models were applied where odds ratios (ORs) and 95% confidence intervals (CIs) were estimated for atopy on the risk of asthma, rhinitis or eczema, respectively. Models were adjusted for age, gender, body mass index (BMI), household passive smoking, and season of birth, which were similar to previous studies^17^. We calculated the PAR to represent the percentage of allergic diseases (i.e. asthma, rhinitis or eczema) contributed by atopy or each category of allergen-specific IgEs. Assuming that allergen categories were mutually independent, we add one allergen category at a time to the models. The formula was shown as PAR = p (RR-1)/(RR), where p is the proportion of population exposed and RR is the adjusted estimate of relative risk of the exposed vs the unexposed. ORs were used to approximate RR as suggested by the previous study^17,29^. The confidence intervals for the PARs were calculated with a logit transformation to constraint the PARs estimation between 0 to 100%. A LOGISTIC procedure in SAS was utilized to estimate PARs and of 95% CIs adjusting for the potential confounding factors^30,31^. SAS version 9.4 for Windows (SAS Institute, Cary, NC) was utilized for statistical analyses in the present study. A two-side p-value of < 0.05 was considered as statistically significant.

## Results

### Subject characteristics

Among the 1,321 subjects, 644 of them (48.8%) are boys and the mean age was 10.3 years (standard deviation 2.7; range 5–18). The prevalence of physician-diagnosed asthma, rhinitis and eczema was 11.2%, 37.7% and 19.5%, respectively (Table 1). The prevalence of atopy among subjects in this study was 57.3%. The prevalence of physician-diagnosed asthma, rhinitis and eczema among atopic subjects was 15.2%, 48.8% and 25.4%, respectively, while the corresponding prevalence among non-atopic subjects was 5.9%, 22.9%, and 11.7%, respectively. The prevalence of physician-diagnosed allergic diseases were significantly higher among atopic than non-atopic subjects (all p < 0.001). Atopic subjects tended to have higher proportions of boys (54.2% vs 41.5%, p < 0.001) and parental allergic diseases (51.3% vs 41.3%, p = 0.002) compared to non-atopic subjects.

**Table 1:** Characteristics of study subjects.

	**All subjects**	**Atopy**	**Non atopy**	**p**
Age, years (mean ± SD)	10.3 ± 2.7	10.3 ± 2.7	10.4 ± 2.7	0.625
Gender, male, n (%)	644/1321 (48.8%)	410/757 (54.2%)	234/564 (41.5%)	< 0.001*
Body mass index, kg/m^2^ (mean ± SD)	18.7 ± 3.6	18.8 ± 3.7	18.5 ± 3.4	0.087
**Body mass index category**				0.199
Underweight, n (%)	107/1321 (8.1%)	53/757 (7.0%)	54/564 (9.6%)	
Normal weight, n (%)	858/1321 (65.0%)	491/757 (64.9%)	367/564 (65.1%)	
Overweight, n (%)	271/1321 (20.5%)	158/757 (20.9%)	113/564 (20.0%)	
Obesity, n (%)	85/1321 (6.4%)	55/757 (7.3%)	30/564 (5.3%)	
Asthma, n (%)	146/1304 (11.2%)	113/743 (15.2%)	33/561 (5.9%)	< 0.001*
Rhinitis, n (%)	487/1293 (37.7%)	360/738 (48.8%)	127/555 (22.9%)	< 0.001*
Eczema, n (%)	253/1297 (19.5%)	188/741 (25.4%)	65/556 (11.7%)	< 0.001*
Parental allergic diseases, n (%)	462/986 (46.9%)	282/550 (51.3%)	180/436 (41.3%)	0.002*
Paternal allergic diseases, n (%)	319/972 (32.8%)	192/542 (35.4%)	127/430 (29.5%)	0.052
Maternal allergic diseases, n (%)	253/974 (26.0%)	163/541 (30.1%)	90/433 (20.8%)	0.001*
Household passive smoking, n (%)	696/1284 (54.2%)	402/736 (54.6%)	294/548 (53.7%)	0.730
**Season of birth**				0.884
Spring, n (%)	312/1321 (23.6%)	183/757 (24.2%)	129/564 (22.9%)	
Summer, n (%)	348/1321 (26.3%)	194/757 (25.6%)	154/564 (27.3%)	
Autumn, n (%)	349/1321 (26.4%)	199/757 (26.3%)	150/564 (26.6%)	
Winter, n (%)	312/1321 (23.6%)	181/757 (23.9%)	131/564 (23.2%)	

*SD* standard deviation.

*P < 0.05 is bold.

### Population risk of allergic diseases attributable to atopy

The PARs of atopy for asthma, rhinitis and eczema were 50.4%, 46.3% and 46.7%, respectively, after adjusting for age, gender, BMI, household passive smoking, and season of birth (Table 2). The PAR of atopy for three allergic diseases was higher in older children aged equal to or more than 10 years (asthma, 54.4%; rhinitis, 59.6%; eczema, 49.5%) than younger children aged less than 10 years (asthma, 46.9%; rhinitis, 39.5%; eczema, 41.9%). In addition, the PAR of atopy for asthma (56.2% vs. 43.3%) and eczema (49.4% vs. 43.2%) was higher among boys than girls, while the PAR of atopy for rhinitis was lower among boys than girls (36.5% vs. 51.1%) (all p < 0.001). Interestingly, while the PAR of atopy for asthma was higher in the obese children (73.3%) than those in the other BMI groups (29.2 to 54.0%), PAR of atopy for eczema was higher in the underweighted children (57.8%) than those in the other BMI groups (34.9 to 49.2%). Subjects with allergic parents had higher PARs for all three allergic diseases (53.3 to 71.8% vs. 19.3 to 33.7%) (Table 2).

**Table 2:** Percentage of allergic diseases attributable to atopy.

	Asthma					Rhinitis					Eczema				
	n	Asthma (%)	Percentage of atopy in asthma cases	aOR* (95% CI)	PAR (95% CI) (%)	n	Rhinitis (%)	Percentage of atopy in rhinitis cases	aOR* (95% CI)	PAR (95% CI) (%)	n	Eczema (%)	Percentage of atopy in eczema cases	aOR* (95% CI)	PAR (95% CI) (%)
Overall	1304	11.2%	77.4%	3.1 (1.7, 4.5)	50.4 (39.2, 61.7)	1293	37.7%	73.9%	3.8 (2.5, 5.1)	46.3 (38.8, 53.7)	1297	19.5%	74.3%	3.1 (2.0, 4.3)	46.7 (37.7, 55.6)
**Age**															
≥ 10 years	671	11.0%	78.4%	3.6 (1.2, 6.0)	54.4 (39.6, 69.3)	667	36.9%	75.6%	5.0 (2.4, 7.7)	59.6 (NC, NC)	665	18.4%	73.8%	3.7 (1.7, 5.6)	49.5 (38.1, 60.9)
< 10 years	633	11.4%	76.4%	2.8 (1.1, 4.5)	46.9 (30.0, 63.8)	626	38.5%	72.2%	3.1 (1.5 4.6)	39.5 (27.4, 51.6)	632	20.7%	74.8%	2.6 (1.4, 3.9)	41.9 (28.0, 55.8)
**Gender**															
Male	634	13.1%	83.1%	3.4 (1.2, 5.6)	56.2 (40.0, 72.4)	632	45.4%	75.6%	2.6 (1.2, 4.0)	36.5 (21.9, 51.0)	629	23.1%	79.3%	3.1 (1.5, 4.7)	49.4 (35.6, 63.1)
Female	670	9.4%	69.8%	2.8 (1.1, 4.6)	43.3 (27.6, 59.0)	661	30.3%	71.5%	45.0 (2.6, 7.3)	51.1 (43.3, 59.0)	668	16.2%	67.6%	3.2 (1.5, 4.8)	43.2 (31.7, 54.6)
**Body mass index category**															
Underweight	107	4.7%	60.0%	2.0 (NC, 7.4)	29.2 (NC, 97.4)	103	28.2%	72.4%	3.9 (NC, 9.8)	51.8 (NC, NC)	104	16.4%	76.5%	4.3 (NC, 11.9)	57.8 (NC, NC)
Normal weight	849	10.3%	79.3%	3.4 (1.5, 5.4)	54.0 (40.6, 67.4)	844	36.1%	74.1%	4.5 (2.6, 6.4)	49.7 (41.5, 57.9)	850	17.7%	75.3%	3.4 (1.9, 4.9)	49.2 (38.7, 59.6)
Overweight	265	14.7%	71.8%	2.5 (0.3, 4.7)	42.7 (NC, NC)	262	42.8%	72.3%	3.3 (0.4, 6.3)	49.2 (NC, NC)	259	23.2%	73.3%	2.8 (0.6, 4.9)	45.9 (NC, NC)
Obesity	83	18.1%	86.7%	7.1 (NC, 22.7)	73.3 (6.4, NC)	84	48.8%	78.1%	3.3 (NC, 9.4)	50.5 (NC, NC)	84	31.0%	69.2%	2.1 (NC, 6.0)	34.9 (NC, NC)
**Parental allergic diseases**															
Yes	457	14.2%	84.6%	6.9 (1.1, 12.7)	71.8 (23.6, NC)	454	47.6%	75.9%	3.8 (1.3, 6.3)	53.3 (NC, NC)	455	25.9%	80.5%	5.7 (1.7, 9.7)	65.3 (NC, NC)
No	518	8.3%	65.1%	2.2 (0.4, 3.9)	33.7 (8.7, 58.6)	513	29.2%	67.3%	2.1 (0.9, 3.3)	30.8 (12.9, 48.8)	516	15.1%	64.1%	1.5 (0.6, 2.4)	19.3 (NC, 66.6)
**Household passive smoking**															
Yes	684	13.0%	76.4%	2.7 (1.2, 4.2)	45.8 (29.9, 61.6)	680	36.5%	75.4%	3.5 (1.9, 5.2)	47.5 (36.6, 58.4)	680	16.6%	77.0%	3.1 (1.4, 4.7)	48.2 (34.0, 62.5)
No	585	9.6%	78.6%	4.1 (1.0, 7.2)	57.5 (42.2, 72.7)	578	39.8%	72.6%	4.4 (2.1, 6.7)	46.1 (36.2, 56.0)	583	23.0%	73.1%	3.1 (1.6, 4.7)	49.1 (NC, NC)
**Season of birth**															
Spring	310	12.6%	76.9%	3.3 (0.5, 6.1)	50.1 (29.4, 70.8)	306	37.6%	73.0%	3.9 (0.9, 6.9)	51.6 (NC, NC)	308	18.8%	75.9%	3.4 (0.9, 5.8)	51.7 (NC, NC)
Summer	341	10.3%	71.4%	3.2 (0.4, 6.1)	49.3 (18.4, 80.2)	334	36.8%	71.5%	4.4 (1.1, 7.6)	47.4 (33.6, 61.2)	340	19.4%	69.7%	4.1 (1.1, 7.2)	49.4 (35.3, 63.5)
Autumn	342	12.3%	83.3%	4.6 (0.54 8.6)	64.9 (26.5, NC)	345	40.3%	74.1%	3.04 (1.0, 5.1)	41.0 (24.3, 57.6)	340	18.2%	74.2%	2.4 (0.6, 4.2)	39.6 (16.4, 62.8)
Winter	311	9.7%	76.7%	2.4 (NC, 5.0)	43.3 (9.8, 76.9)	308	35.7%	77.3%	6.8 (1.2, 12.5)	65.0 (NC, NC)	309	21.7%	77.6%	3.6 (0.9, 6.4)	55.5 (NC, NC)

*aOR* adjusted odds ratio, *CI* confidence interval, *PAR* population attributable risk, *NC* not computed.

*Adjusted for age, gender, body mass index, household passive smoking, and season of birth.

We further performed stratified analysis by gender and age (Table 3). The results suggested that allergic diseases were more likely attributed to atopy among older children equal to or more than 10 years than younger children aged less than 10 years in both boys and girls.

**Table 3:** Percentage of allergic diseases attributable to atopy, stratified by gender and age.

	Male				Female			
	n	Percentage of atopy	aOR* (95%CI)	PAR (95% CI) (%)	n	Percentage of atopy	aOR* (95%CI)	PAR (95% CI) (%)
**Asthma**								
Age ≥ 10 years	313	85.7%	4.1 (1.6, 10.2)	62.2 (NC, NC)	358	68.8%	2.1 (0.9, 4.6)	46.2 (25.6, 66.7)
Age < 10 years	321	80.5%	3.2 (1.4, 7.2)	53.2 (29.9, 76.6)	312	71.0%	2.8 (1.2, 6.4)	42.3 (NC, 96.8)
**Rhinitis**								
Age ≥ 10 years	312	77.7%	3.4 (2.0, 5.7)	52.7 (NC, NC)	355	72.9%	4.0 (2.4, 6.8)	63.2 (NC, NC)
Age < 10 years	320	73.7%	2.5 (1.6, 4.2)	38.9 (16.8, 61.0)	306	69.9%	2.8 (1.7, 4.9)	41.1 (27.3, 54.9)
**Eczema**								
Age ≥ 10 years	309	80.8%	3.2 (1.6, 6.2)	58.0 (NC, NC)	356	63.3%	2.0 (1.1, 4.0)	42.1 (27.6, 56.6)
Age < 10 years	320	77.8%	2.7 (1.4, 5.1)	43.3 (21.9, 64.7)	312	71.2%	3.0 (1.6, 5.6)	39.4 (21.0, 57.7)

*aOR* adjusted odds ratio, *CI* confidence interval, *PAR* population attributable risk, *NC* not computed.

*Adjusted for age, gender, body mass index, household passive smoking, and season of birth.

### Allergen-specific IgE and allergic diseases

Table 4 shows the associations between allergen-specific IgEs and allergic diseases. Sensitization to mites had the highest PAR for all three allergic diseases (asthma, 64.1%; rhinitis, 51.3%; eczema, 57.2%) in the current study, followed by sensitization to foods (asthma, 7.1%; rhinitis, 10.4%; eczema, 27.7%). The PAR of mite sensitization was higher among subjects with asthma than those with rhinitis or eczema. The PAR of food sensitization in explaining eczema is 27.7%, which was higher than those in explaining rhinitis (10.4%) or asthma (7.1%).

**Table 4:** Percentage of allergic diseases attributable to allergen-specific sensitization.

Category of allergen*	Asthma		Rhinitis		Eczema	
	aOR (95% CI)	PAR (95% CI) (%)	aOR* (95% CI)	PAR (95% CI) (%)	aOR* (95% CI)	PAR (95% CI) (%)
Mites	4.1 (1.7, 6.4)	64.1 (50.8, 77.3)	3.9 (2.4, 5.4)	51.3 (42.1, 60.5)	3.6 (2.1, 5.2)	57.2 (46.2, 68.2)
Foods	1.1 (0.7, 1.6)	7.1 (NC, 28.5)	1.3 (0.9, 1.7)	10.4 (NC, 22.1)	1.8 (1.2, 2.4)	27.7 (14.3, 41.1)
Mold	1.2 (0.7, 1.7)	4.0 (NC, 12.7)	0.5 (0.3, 0.6)	†	1.0 (0.6, 1.4)	0.7 (NC, 9.1)
Animals	1.3 (0.6, 2.0)	3.4 (NC, 9.3)	0.5 (0.3, 0.7)	†	1.2 (0.7, 1.7)	2.5 (NC, 8.3)
Cockroaches	1.1 (0.5, 1.6)	1.3 (NC, 9.2)	0.9 (0.5, 1.2)	†	0.7 (0.4, 1.0)	†
Pollen	0.6 (0.3, 0.9)	†	0.6 (0.4, 0.8)	†	1.0 (0.6, 1.3)	†
Latex	‡	‡	‡	‡	‡	‡

*aOR* adjusted odds ratio, *CI* confidence interval, *PAR* population attributable risk, *NC* not computed.

*Each allergen category was independently analyzed.

^†^PAR were not calculated because the aOR was < 1.0^17^.

^‡^aOR and PAR were not calculated for latex due to its low prevalence which resulted in non-convergence of the model^28^.

## Discussion

This study demonstrates several main findings. First, 50.4% of asthma, 46.3% of rhinitis and 46.7% of eczema cases were attributable to atopy in this population-based sample of 1321 Asian school-age children. Second, allergic diseases were more likely attributed to atopy among adolescents compared to younger children. Third, the PAR of atopy for asthma and eczema was significantly higher among boys than girls, while the PAR of atopy for rhinitis was lower among boys than girls. Fourth, among the seven allergen categories, sensitization to mites had the highest PAR for all three allergic diseases (51.3% to 64.1%), followed by sensitization to foods (PAR of 27.7% for eczema, 10.4% for rhinitis and 7.1% for asthma).

Asthma, rhinitis and eczema were generally considered atopic diseases associated with specific IgE antibodies while also co-occur in non-sensitized individuals^32^. This study demonstrates that the estimated PAR of atopy for allergic diseases among Asian school-age children ranged from 46.3 to 50.4%. As PAR is a parameter signaling the proportion of diseases which may be preventable by eliminating the exposure to allergens^33,34^, our findings imply that approximately half of the children with allergic diseases may potentially benefit from avoidance of culprit allergen(s). However, the other half of children with asthma, rhinitis and eczema may have a non-atopic etiology which is unlikely to benefit from aggressive allergen avoidance. There remains an ongoing need for further exploration in etiologies and management strategies for a significant proportion of children with allergic diseases attributable to non-IgE-mediated mechanisms.

Approximately half (46.3 to 50.4%) of Asian children with allergic diseases in this study were attributable to atopy, which falls in the range of the existing references toward the higher end^10–12,16–18^. Limited data is available regarding the proportion of allergic diseases attributable to atopy among Asian children. Previous studies reported that the proportion of rhinitis children attributable to atopy ranged from 20.6 to 39.1% in three south-east Asian populations^12,35^. While ethnic disparities may partly explain the differences in prevalence of atopy as well as allergic diseases^14,15^, it remains possible that differences regarding the definitions of atopy and allergic diseases, environmental exposures, and socioeconomic and lifestyle factors can also influence the estimated PARs of atopy^12,16,17^. For example, the distribution of PAR of atopy for asthma ranged from 8 to 63% across studies when atopy is defined as at least one positive skin prick test^10,11,17,18^. On the other hand, when atopy was defined as the presence of serum allergen-specific IgE to any of 5 allergens, Sunyer et al.^16^ reported that the proportion of asthma attributable to atopy was around 30% but varied widely from 4 to 61% across 36 centers in 16 European countries. Similar scenarios were demonstrated in cases with rhinitis and eczema. Zacharasiewicz et al.^12^ reported that the attributable risk of atopy in children with physician-diagnosed hay fever ranged widely from 37.3 to 80.7% in 22 population-based studies. Even among populations of the same ethnicity, the geographic variations and center differences resulted in a wide distribution of attributable risk of atopy for asthma ranging from 9 to 61% in Spain^16^. Therefore, currently available data do not provide sufficient information to reach a conclusion about the influence of ethnic factors on the population risk of allergic diseases attributable to atopy.

Our data demonstrated that allergic diseases, particularly allergic rhinitis, were more likely attributed to atopy among adolescents compared to younger children. Further studies are needed to validate our findings and to explore potential mechanisms. In the Isle of Wight birth cohort, Kurukulaaratchy et al.^36^ demonstrated that atopic rhinitis which was defined as both rhinitis and allergic sensitization became increasingly common as children grow into adolescents. It seems reasonable to postulate that increasing aeroallergen sensation during the first decade of life probably perpetuate a state of tissue inflammation responsible for the development of atopic rhinitis from childhood to adolescence.

The gender differences of PAR for allergic diseases is an interesting observation in the present study, although the findings should be interpreted with caution. Whereas boys were more likely to have asthma, rhinitis and eczema than girls, our findings suggest that boys were more likely to have atopic asthma and atopic eczema while girls were more likely to have atopic rhinitis. Using data from the Third National Health and Nutrition Examination Survey, Arbes et al.^17^ also found that boys are more likely to have atopic asthma than girls. Although the mechanism for the differences in PAR by gender remains unknown, potential explanations for the differences in PAR by gender may include differences in reproductive hormones, environmental exposures and lifestyle factors.

Of the seven categories of allergens tested, mite sensitization was the leading factor attributed to asthma, rhinitis and eczema after adjusting for subjects’ characteristics in the present study. Previous studies reported remarkably high prevalence of mite sensitization, 80 to 90%, in several Asia countries including Taiwan, Korea, Indonesia, India and China^37^. Our study identified mite sensitization as a major attributable cause of allergic diseases in Taiwan, a subtropical island country with warm and humid environment. In addition, one-fourth of the children with eczema in this study can be attributable to food sensitization. The PAR of food sensitization for children with eczema is much higher than that for children with allergic airway diseases. In line with previous studies, these results suggest that avoiding the ingestion of allergenic foods may help to reduce allergic skin conditions but have limited impact on allergic airway diseases^10,38,39^.

The strengths of this population-based study of 1321 Asian children aged 5 to 18 years include a large sample size, a representative community sampling, and the measurement of a wide spectrum of allergen-specific IgEs. This study has some limitations. First, the cross-sectional design of this study precludes the possibility to establish the temporal relationship between atopy and allergic diseases. This limitation, however, is common to most published articles examining the percentage of allergic diseases attributable to atopy. Second, the attributable risk of atopy may vary across countries as a result of variations in exposure. Therefore our findings should be considered in the context of Taiwan and may not be generalizable to other Asian countries. Third, diagnostic misclassification is a potential concern for studies relied on physician diagnosis of allergic diseases. The effect of diagnostic misclassification on evaluating the PAR of atopy is unclear and needs further investigation.

In conclusion, our study suggests that approximately half (46.3 to 50.4%) of Asian children in Taiwan with allergic diseases are attributable to atopy, implying that the other half of these cases might be accounted for by organ-based and/or other factors rather than allergic sensitization. Our study also highlights the need for further research into the causes of allergic diseases mediated by non-IgE-mediated mechanisms.

## Abbreviations

BMI: Body mass index
CI: Confidence interval
IgE: Immunoglobulin E
ISAAC: International Study of Asthma and Allergies in Childhood
NC: Not computed
OR: Odds ratio
PAR: Population attributable risk
PATCH: Prediction of Allergies in Taiwanese CHildren
RR: Relative risk
SD: Standard deviation

## References

[1] Asher MI (2006). Worldwide time trends in the prevalence of symptoms of asthma, allergic rhinoconjunctivitis, and eczema in childhood: ISAAC Phases One and Three repeat multicountry cross-sectional surveys. Lancet.

[2] Worldwide variation in prevalence of symptoms of asthma, allergic rhinoconjunctivitis, and atopic eczema: ISAAC. The International Study of Asthma and Allergies in Childhood (ISAAC) Steering Committee. *Lancet* **351**, 1225–1232, (1998). 9643741

[3] Yao TC (2011). Associations of age, gender, and BMI with prevalence of allergic diseases in children: PATCH study. J. Asthma.

[4] Silverberg JI (2015). Health care utilization, patient costs, and access to care in US adults with eczema: A population-based study. JAMA Dermatol..

[5] Jacob C (2016). Healthcare costs and resource utilization of asthma in Germany: A claims data analysis. Eur. J. Health Econ..

[6] Callander EJ, Schofield DJ (2015). Effect of asthma on falling into poverty: The overlooked costs of illness. Ann. Allergy Asthma Immunol..

[7] Blaiss MS (2000). Cognitive, social, and economic costs of allergic rhinitis. Allergy Asthma Proc..

[8] Carroll CL, Balkrishnan R, Feldman SR, Fleischer AB, Manuel JC (2005). The burden of atopic dermatitis: Impact on the patient, family, and society. Pediatr. Dermatol..

[9] Ballardini N (2016). IgE antibodies in relation to prevalence and multimorbidity of eczema, asthma, and rhinitis from birth to adolescence. Allergy.

[10] Arshad SH, Tariq SM, Matthews S, Hakim E (2001). Sensitization to common allergens and its association with allergic disorders at age 4 years: A whole population birth cohort study. Pediatrics.

[11] Pearce N, Pekkanen J, Beasley R (1999). How much asthma is really attributable to atopy?. Thorax.

[12] Zacharasiewicz A, Douwes J, Pearce N (2003). What proportion of rhinitis symptoms is attributable to atopy?. J. Clin. Epidemiol..

[13] Burrows B, Martinez FD, Halonen M, Barbee RA, Cline MG (1989). Association of asthma with serum IgE levels and skin-test reactivity to allergens. N. Engl. J. Med..

[14] Hjern A, Haglund B, Hedlin G (2000). Ethnicity, childhood environment and atopic disorder. Clin. Exp. Allergy.

[15] Joseph CL, Williams LK, Ownby DR, Saltzgaber J, Johnson CC (2006). Applying epidemiologic concepts of primary, secondary, and tertiary prevention to the elimination of racial disparities in asthma. J. Allergy Clin. Immunol..

[16] Sunyer J (2004). Geographic variations in the effect of atopy on asthma in the European Community Respiratory Health Study. J. Allergy Clin. Immunol..

[17] Arbes SJ, Gergen PJ, Vaughn B, Zeldin DC (2007). Asthma cases attributable to atopy: Results from the Third National Health and Nutrition Examination Survey. J. Allergy Clin. Immunol..

[18] Ponsonby AL (2002). Which clinical subgroups within the spectrum of child asthma are attributable to atopy?. Chest.

[19] Yao TC (2011). Exhaled nitric oxide discriminates children with and without allergic sensitization in a population-based study. Clin. Exp. Allergy.

[20] Yao TC (2012). Reference values of exhaled nitric oxide in healthy Asian children aged 5 to 18 years. Eur. Respir. J..

[21] Yao TC (2016). Tobacco smoke exposure and multiplexed immunoglobulin E sensitization in children: A population-based study. Allergy.

[22] Yao TC (2017). Obesity disproportionately impacts lung volumes, airflow and exhaled nitric oxide in children. PLoS ONE.

[23] Yao TC (2019). Genetic loci determining total immunoglobulin E levels from birth through adulthood. Allergy.

[24] Chang SM (2019). Reference equations for spirometry in healthy Asian children aged 5 to 18 years in Taiwan. World Allergy Organ J..

[25] Asher MI (1995). International study of asthma and allergies in Childhood (ISAAC): Rationale and methods. Eur. Respir. J..

[26] Ballardini N, Nilsson C, Nilsson M, Lilja G (2006). ImmunoCAP Phadiatop Infant–a new blood test for detecting IgE sensitisation in children at 2 years of age. Allergy.

[27] Yao TC (2014). Multiplexed immunoglobulin E sensitization in relation to exhaled nitric oxide in a population sample of children. Allergy.

[28] Soriano JB (1999). Risk of asthma in the general Spanish population attributable to specific immunoresponse. Spanish Group of the European Community Respiratory Health Survey. Int. J. Epidemiol..

[29] Fleiss JL, Levin B, Paik MC (2003). Statistical Methods for Rates and Proportions.

[30] Benichou J (1991). Methods of adjustment for estimating the attributable risk in case-control studies: A review. Stat. Med..

[31] Greenland S (1987). Variance estimators for attributable fraction estimates consistent in both large strata and sparse data. Stat. Med..

[32] Garcia-Aymerich J (2015). Phenotyping asthma, rhinitis and eczema in MeDALL population-based birth cohorts: An allergic comorbidity cluster. Allergy.

[33] Mansournia MA, Altman DG (2018). Population attributable fraction. BMJ.

[34] Miettinen OS (1974). Proportion of disease caused or prevented by a given exposure, trait or intervention. Am. J. Epidemiol..

[35] Leung R, Ho P (1994). Asthma, allergy, and atopy in three south-east Asian populations. Thorax.

[36] Kurukulaaratchy RJ (2011). The influence of gender and atopy on the natural history of rhinitis in the first 18 years of life. Clin. Exp. Allergy.

[37] Acevedo N, Zakzuk J, Caraballo L (2019). House dust mite allergy under changing environments. Allergy Asthma Immunol. Res..

[38] Caubet JC, Eigenmann PA (2010). Allergic triggers in atopic dermatitis. Immunol. Allergy Clin. N. Am..

[39] Roberts G, Golder N, Lack G (2002). Bronchial challenges with aerosolized food in asthmatic, food-allergic children. Allergy.

